# NOP56 interacts with Fibrarin to regulate the PI3K/AKT signaling pathway and inhibit apoptosis of hepatocellular carcinoma

**DOI:** 10.3389/fonc.2025.1728226

**Published:** 2026-01-06

**Authors:** Hongwei Chen, Xinggang Fan, Di Cui

**Affiliations:** 1 Fuyang Medical College, Fuyang Normal University, Fuyang, Anhui, China; 2 Traditional Chinese Medicine Department, Fuyang Normal University Affiliated Second Hospital, Fuyang, Anhui, China

**Keywords:** apoptosis, fibrillarin, hepatocellular carcinoma, NOP56, PI3K/AKT/CREB

## Abstract

**Introduction:**

Hepatocellular carcinoma (HCC) is a major cause of cancer-related mortality. While *C-myc* is known to drive hepatocarcinogenesis, the roles of its downstream targets remain unclear. NOP56, a conserved nucleolar protein and *C-myc* target, may contribute to HCC progression.

**Methods:**

We analyzed single-cell and bulk transcriptomic datasets to determine NOP56 expression and clinical significance. Loss-of-function assays in HCC cells, along with xenograft models, were used to evaluate its biological role. Protein interaction and pathway analyses were conducted using co-immunoprecipitation and Western blotting.

**Results:**

NOP56 was upregulated in malignant hepatocytes and associated with poor prognosis. NOP56 knockdown inhibited proliferation, colony formation, and migration, induced G0/G1 arrest and apoptosis, and reduced tumor growth in vivo. Mechanistically, NOP56 interacted with fibrillarin (FBL) and activated the PI3K/AKT/CREB pathway. Silencing NOP56 lowered FBL levels and suppressed pathway activity, whereas FBL overexpression partially rescued apoptotic effects.

**Discussion:**

NOP56 promotes HCC progression through the NOP56–FBL–PI3K/AKT/CREB axis. These findings reveal a previously unrecognized oncogenic role of nucleolar proteins in HCC and highlight this signaling axis as a promising therapeutic target.

## Introduction

Hepatocellular carcinoma (HCC) causes about 865,000 new cases and 758,000 deaths worldwide each year, reflecting its heavy health burden (1, 2). Because early-stage HCC often lacks clear symptoms, including jaundice, weight loss, ascites, or right upper quadrant discomfort, most patients are diagnosed at intermediate stages (3). Although recent advancements in surgical resection, local ablative therapies, targeted agents, and immune checkpoint inhibitors have improved survival in select patient populations, the overall 5-year survival rate for advanced-stage HCC remains below 20% (4). One of the critical challenges in clinical management is the lack of highly specific and effective therapeutic targets, which contribute to frequent treatment resistance and tumor recurrence (5). Therefore, a deeper understanding of the molecular mechanisms driving hepatocarcinogenesis is essential for identifying novel therapeutic strategies.

At the molecular level, HCC arises through a multistep process characterized by chronic inflammation, fibrosis, cirrhosis, and the gradual accumulation of genetic and epigenetic alterations (6). Several major oncogenic pathways—including Wnt/β-catenin, PI3K/AKT/mTOR, RAS/MAPK, JAK/STAT, and TGF-β signaling—cooperate to promote uncontrolled proliferation, metabolic reprogramming, resistance to apoptosis, and metastatic progression (7). In addition to these classical pathways, recent studies highlight that dysregulated ribosome biogenesis, RNA modification, and chromatin remodeling also play critical roles in sustaining malignant transformation, indicating that nucleolar processes and transcriptional reprogramming are emerging hallmarks of HCC (8).

C-Myc is a key oncogene driving HCC initiation, progression, and spread (9, 10). Studies show its mRNA and protein levels are notably increased in HCC, and abnormal expression is linked to poor prognosis (11–13). As a transcription factor, C-myc regulates genes involved in cell cycle control, differentiation, adhesion, and transformation (14, 15). Beyond these classical functions, C-Myc reprograms cellular metabolism by promoting glycolysis and glutamine utilization, enhances ribosome biogenesis, induces DNA replication stress, and globally amplifies oncogenic transcriptional programs (16, 17). Moreover, C-Myc frequently interacts with major HCC-related signaling pathways such as Wnt/β-catenin and PI3K/AKT, forming feed-forward loops that further accelerate tumorigenesis and malignant progression (18, 19).

Nucleolar protein 5A (NOP56), one of the target genes of C-myc, encodes a highly conserved nucleolar protein from yeast to humans (20). Functionally, NOP56 is a core component of the box C/D small nucleolar ribonucleoprotein (snoRNP) complex, participating in rRNA 2’-O-methylation, pre-rRNA processing, and ribosome assembly. Growing evidence suggests that nucleolar proteins such as NOP56 influence tumor progression not only through ribosome biogenesis but also by regulating cellular stress responses and oncogenic signaling pathways (21). NOP56 is overexpressed in several cancers, such as lung adenocarcinoma (22), acute lymphoblastic leukemia (23), and breast cancer (24). Its silencing increases ROS levels and induces apoptosis in KRAS-mutant cells (25). However, the specific functional role and mechanistic contribution of NOP56 in HCC remain largely unknown. Fibrillarin (FBL), another essential box C/D snoRNP protein, is responsible for rRNA 2’-O-methylation and the maturation of 18S and 28S rRNA (26). Elevated FBL expression has been reported in multiple cancers, where it promotes ribosome biogenesis, enhances proliferation, and supports oncogenic translational programs (27). Although FBL has been linked to tumor aggressiveness and metabolic reprogramming, its involvement in hepatocarcinogenesis has not been fully elucidated.

However, the functional role and underlying mechanisms of NOP56 and FBL in HCC remain unclear. We investigated NOP56 in HCC using bioinformatics and molecular experiments to clarify its role and therapeutic potential. These findings aim to provide a basis for identifying novel precision therapeutic targets for HCC.

## Methods

### Data acquisition and preprocessing

Single-cell RNA-seq data for HCC (GSE166635) were downloaded from GEO and processed in R (Seurat v4.0) for quality control, normalization, and dimensionality reduction. Cells with <200 detected genes or >10% mitochondrial reads were removed. Bulk RNA-seq and clinical data for HCC were obtained from the TCGA-LIHC cohort via UCSC Xena. Gene expression values were normalized to FPKM, log_2_-transformed, and used for subsequent analyses. The prognostic genes used in this study were identified from TCGA-LIHC clinical data using univariate Cox regression analysis (P < 0.05), and the resulting gene list is provided in **Supplementary Table 1**. The MYC_TARGETS_V1/V2 gene set was obtained from the Molecular Signatures Database. The complete list of c-Myc target genes used for analysis is provided in **Supplementary Table 2**.

### Single-cell clustering and gene expression analysis

Normalized data underwent PCA and UMAP for visualization, and clusters were annotated using canonical markers. C-myc target gene expression differences among cell types were evaluated with the Wilcoxon rank-sum test.

### Unsupervised clustering and heatmap visualization

Consensus clustering of myc-DEG expression profiles was performed with “ConsensusClusterPlus” in R, and heatmaps were generated using “pheatmap” to display expression patterns across clusters.

### Construction of prognostic models using machine learning

In constructing the prognostic model based on cancer gene expression, we adopted a streamlined approach to balance predictive accuracy and interpretability. All input genes were directly used as features without additional selection or dimensionality reduction, and linear modeling was employed to ensure transparency in assessing each gene’s contribution to prognosis. Data preprocessing involved removing missing values and non-tumor samples, converting survival time from days to years, applying z-score normalization across validation datasets, and transforming normalized values using the exponential function to maintain non-negativity. Multiple algorithms, including Lasso regression, elastic net, ridge regression, stepwise Cox regression, and CoxBoost, were implemented with parameter optimization via cross-validation. Risk scores were calculated as the linear combination of gene expression and model coefficients. Model performance was evaluated using time-dependent ROC curves and the mean AUC at 1-, 3-, and 5-year time points. Univariate Cox regression and meta-analysis (inverse variance method) were used to assess prognostic value.

### Sample acquisition

This study included HCC patients treated at Fuyang Normal University Affiliated Second Hospital. A total of 12 matched pairs of tumor and adjacent non-tumor liver tissues were collected during hepatectomy procedures, with 4 pairs used for immunohistochemistry and 8 for RT-PCR and Western blotting. After resection, samples were rinsed in saline, cleared of blood, and preserved in liquid nitrogen. All cases were pathologically confirmed. Ethical approval was obtained from the hospital’s ethics committee.

### Immunohistochemistry

Formalin-fixed, paraffin-embedded HCC and adjacent liver tissues were sectioned at 4 μm, deparaffinized, rehydrated, and subjected to antigen retrieval in citrate buffer (pH 6.0) at 95°C for 20 min. Endogenous peroxidase was quenched with 3% hydrogen peroxide, followed by overnight incubation at 4°C with anti-NOP56 antibody (1:200) and subsequent HRP-conjugated secondary antibody for 1 h. Staining was visualized with DAB and counterstained with hematoxylin. H-scores were independently evaluated by two pathologists according to staining intensity and percentage of positive cells.

### Cell culture

HepG2, Huh7, SKhep1, PLC, and the normal hepatic cell line MIHA were all purchased from Servicebio (Wuhan, China). Specifically, the normal hepatic cell line MIHA, as well as the hepatocellular carcinoma cell lines Huh7, SKhep1, HepG2, and PLC, were acquired from this commercial supplier. All cell lines were cultured in Dulbecco’s Modified Eagle Medium (DMEM, Gibco, USA) supplemented with 10% fetal bovine serum (FBS) and 1% penicillin-streptomycin (100 U/mL penicillin and 100 μg/mL streptomycin).

### Real-time quantitative polymerase chain reaction

Total RNA was isolated using TRIzol reagent (Invitrogen), followed by reverse transcription into cDNA with the PrimeScript RT reagent kit (Takara) incorporating gDNA Eraser to remove genomic DNA contamination. Quantitative real-time PCR (RT-qPCR) was then conducted on a CFX96 detection system (Bio-Rad) using SYBR Green Master Mix (Takara). ACTB served as internal reference genes, and relative expression levels were determined via the 2^−ΔΔCt calculation method. All reactions were performed in triplicate to ensure reproducibility. The primer sequences used are listed in **Supplementary Table 3**.

### Western blotting

The Western blot procedure was performed as described in previous study (28), and the antibodies used are listed in **Supplementary Table 4**. Protein bands were visualized using enhanced chemiluminescence (Servicebio) and quantified with ImageJ. Original blots are presented in **Supplementary Figure 1**. All experiments were independently repeated three times.

### Immunofluorescence

Cells were fixed with 4% paraformaldehyde and permeabilized with 0.1% Triton X-100, followed by blocking with 5% BSA. Samples were incubated with primary antibodies at 4°C overnight and then with fluorophore-conjugated secondary antibodies for 1 h at room temperature. Nuclei were counterstained with DAPI. Images were captured using a Zeiss LSM 880 confocal microscope. Subcellular localization, particularly nucleolar distribution, was evaluated based on fluorescence signals. The procedure was performed according to our previously published protocol (28). All experiments were independently repeated three times.

### Construction of stable NOP56 knockdown cell lines

Two NOP56-targeting shRNAs (sh1 and sh2), together with a non-targeting control (NC), were cloned into the pLKO.1 lentiviral vector (Tsingke, China). The targeting sequences were: shNOP56-1, 5′-ACAGGAGAAGAAACGCTTAAA-3′; shNOP56-2, 5′-CCTGGCAAACAAATGCAGTAT-3′. Lentiviruses carrying these plasmids were used to infect Huh7 and SKhep1 cells in the presence of 8 μg/mL polybrene (Sigma). After 48 h, infected cells were selected with 2 μg/mL puromycin for 7 days before subsequent experiments.

### Evaluation of colony formation, wound healing, and transwell migration

For colony formation, ~500 cells were plated in 6-well plates and cultured for 10–14 days, then fixed, crystal violet–stained, and colonies (>50 cells) counted with ImageJ. Wound-healing assays were performed by scratching confluent monolayers with pipette tips and imaging at 0 and 48 h. Transwell assays used 24-well inserts (8 μm pores): 2×10^4^ cells in serum-free medium were seeded in the upper chamber, with 10% FBS medium below. Migrated cells were fixed, stained, and quantified in five random 200× fields.

### *In vivo* xenograft tumor model

Four- to five-week-old male BALB/c nude mice (initial weight: 16–18 g, ending weight: 22–24 g) were purchased from Qingyuan BioTechnology Co., Ltd.; all procedures complied with institutional guidelines, were approved by the Animal Ethics Committee of Fuyang Normal University and followed ARRIVE guidelines (https://arriveguidelines.org). Prior to subcutaneous injection of Huh7 cells (5×10^6^ cells resuspended in 100 μL sterile PBS, pH 7.4) into the right dorsal flank (27-gauge needle, n=5 per group), mice were anesthetized via intraperitoneal injection of 2% pentobarbital sodium (50 mg/kg), with depth confirmed by absent pedal reflex and 30–40 breaths/min; post-injection, the site was disinfected with 75% ethanol, and mice recovered in a 30–32°C cage (30–45 min, confirmed by voluntary movement/grooming). Tumor size was measured every 3 days (no anesthesia, gentle restraint, digital caliper, 0.01 mm accuracy) and volume calculated as (length×width^2^)/2, with simultaneous body weight recording. After 28 days, mice were euthanized via cervical dislocation post-anesthesia: first, intraperitoneal injection of 2% pentobarbital sodium (150mg/kg, overdose), with deep anesthesia confirmed by absent corneal/pedal/tail pinch reflexes and <10 breaths/min (10-15min), followed by cervical dislocation (firm head/body hold, quick upward traction/lateral rotation); death was confirmed via 2 min of no chest movement (breathing cessation), 1 min of no thoracic heartbeat (palpation), and rechecked absent reflexes. Tumors were then dissected, rinsed with cold sterile PBS, photographed, and weighed for further analysis. The diameters of all tumors are shown in **Supplementary Table 5**.

### Cell cycle and apoptosis assays

For cell cycle analysis, Huh7 and SKhep1 cells (± NOP56 knockdown) were fixed in 70% ethanol at –20°C, stained with PI/RNase A, and analyzed on a BD FACSCanto II cytometer to determine cell cycle distribution using FlowJo. Apoptosis was assessed with the Annexin V-FITC/PI Kit (Beyotime): cells were stained for 15 min at room temperature, followed by flow cytometry within 1 h, and apoptotic fractions quantified with FlowJo.

### Co-immunoprecipitation and silver staining

HCC cells were lysed in IP buffer containing protease inhibitors. Lysates were pre-cleared with Protein A/G beads and incubated overnight at 4°C with anti-NOP56 or anti-FBL antibodies. Immune complexes were captured, washed, and eluted for SDS-PAGE. Gels were silver stained using the Fast Silver Staining Kit (Beyotime), and proteins of interest were validated by reciprocal Western blotting.

### Statistical analysis

Statistical analyses were conducted using R software (v4.2.2) and GraphPad Prism 9.0. A *P*-value<0.05 was considered statistically significant.

## Results

### C-myc target genes define prognostic subtypes and immune patterns in HCC

**Figure 1:**
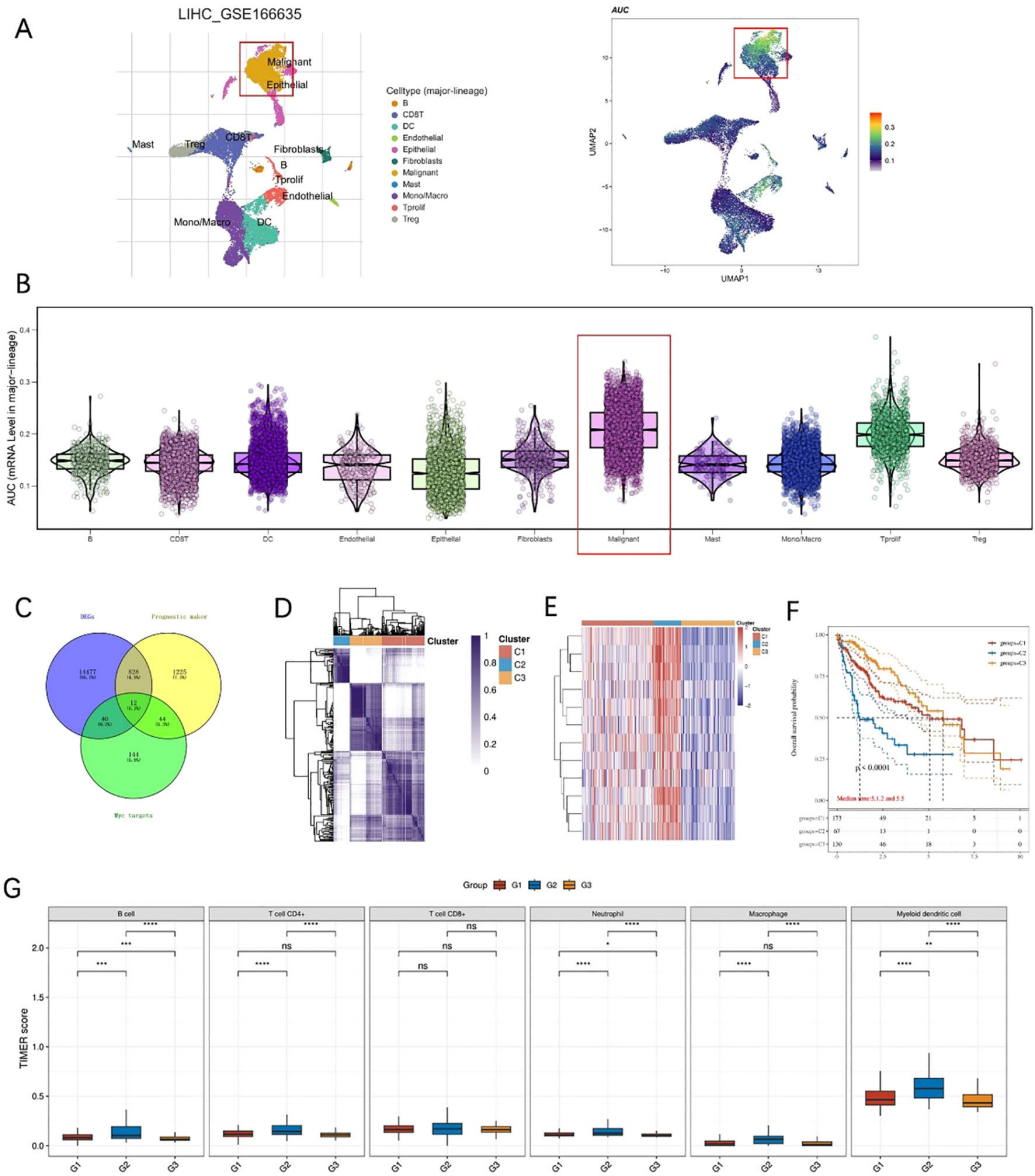
C-myc target genes define molecular subtypes and immune patterns in HCC. **(A)** UMAP plots showing cell type distribution and C-myc target gene activity in single-cell RNA-seq data (GSE166635). **(B)** Violin plots showing higher C-myc target gene expression in malignant hepatocytes. **(C)** Venn diagram identifying 12 myc-related prognostic genes by overlapping DEGs, prognostic markers, and C-myc targets. **(D)** Consensus clustering of TCGA-HCC samples based on myc-DEGs defines three subtypes (C1–C3). **(E)** Heatmap showing expression of myc-DEGs across clusters. **(F)** Kaplan–Meier curves showing poor survival in cluster C2. **(G)** Immune infiltration analysis reveals distinct immune cell compositions among clusters.

GSE166635 is a published single-cell RNA-seq dataset generated from 25,189 primary liver cancer cells from two patients, reported by Meng et al (29). The dataset includes two tumors sequenced using the 10x Genomics platform and publicly released on June 9, 2021. Analysis of the single-cell RNA sequencing dataset GSE166635 revealed distinct cellular subpopulations within HCC (**Figure 1A**). Examination of C-myc target gene expression across different cell types demonstrated markedly elevated expression in malignant hepatocytes compared with non-malignant cell populations (**Figure 1B**). To identify C-myc–related prognostic biomarkers in HCC, differentially expressed genes (DEGs) from the LIHC-TCGA dataset were intersected with prognostic genes and the C-myc target gene set, yielding 12 overlapping genes (myc-DEGs) (**Figure 1C**). Unsupervised clustering of TCGA-HCC patients based on the expression profiles of these 12 myc-DEGs (CDC45, PLK4, TYMS, NME1, CAD, FBL, IMPDH2, NOP56, NHP2, SRM, PRMT3, CNBP) stratified the cohort into three distinct clusters (C1–3) (**Figure 1D**). Heatmap analysis demonstrated that C2 exhibited globally elevated expression levels of these 12 myc-DEGs (**Figure 1D**). Kaplan–Meier survival analysis indicated that patients in C2 had the poorest overall survival compared with C1 and C3 (**Figure 1E**). Furthermore, immune infiltration analysis revealed significant differences in the immune microenvironment among the three clusters, with C2 showing distinct immune cell infiltration patterns (**Figure 1G**). These findings suggest that C-myc–associated transcriptional programs may be linked to tumor aggressiveness and immune landscape alterations in HCC.

### Establishment of prognostic models

**Figure 2:**
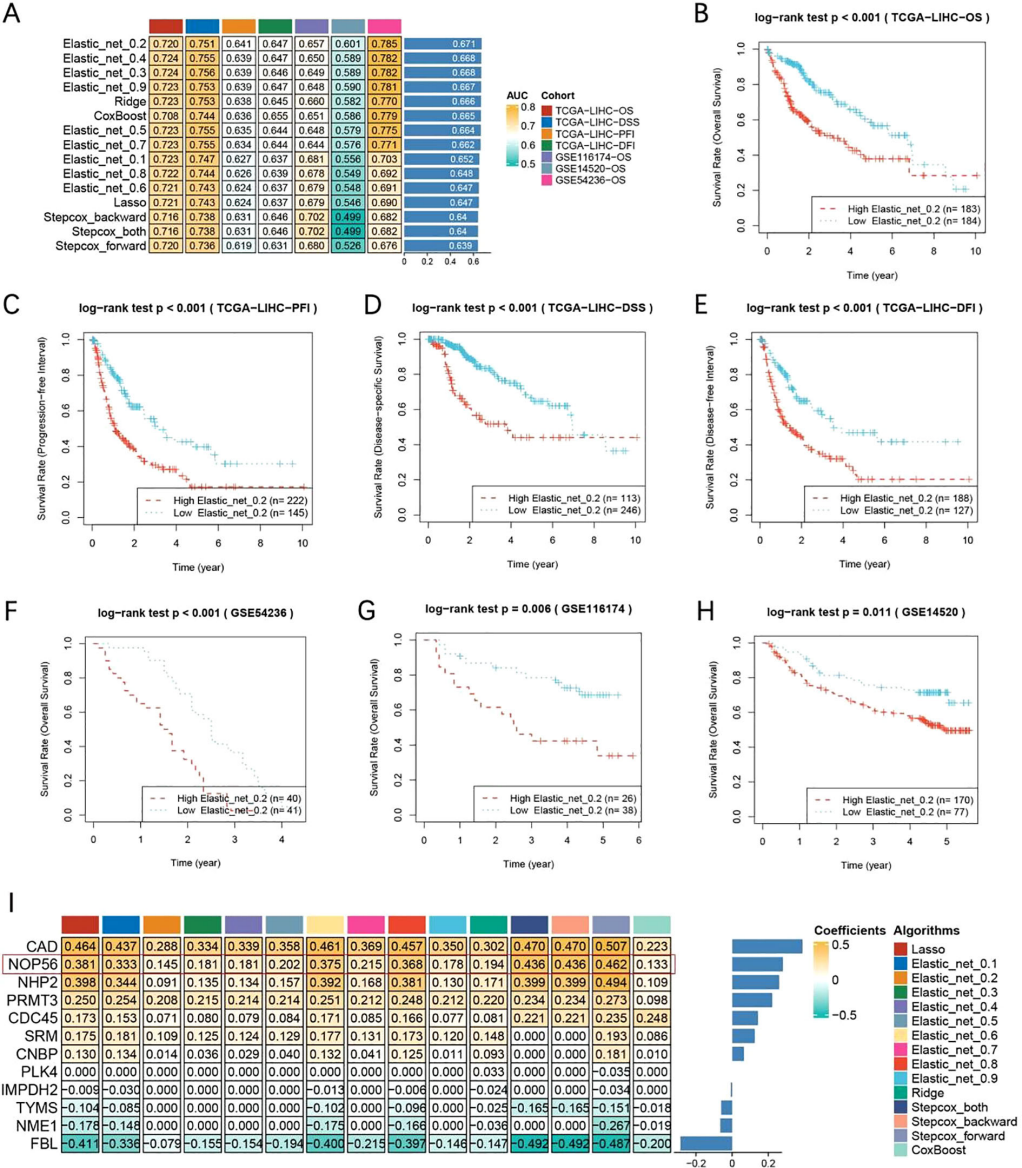
Construction and validation of prognostic models using multiple machine learning algorithms. **(A)** Heatmap showing average AUC values for 1-, 3-, and 5-year survival across seven cohorts and multiple algorithms. **(B)** Kaplan–Meier overall survival analysis in TCGA-LIHC cohort based on the Elastic_net_0.2 model. **(C)** Kaplan–Meier progression-free interval (PFI) analysis in TCGA-LIHC cohort. **(D)** Kaplan–Meier disease-specific survival (DSS) analysis in TCGA-LIHC cohort. **(E)** Kaplan–Meier disease-free interval (DFI) analysis in TCGA-LIHC cohort. **(F)** Kaplan–Meier overall survival analysis in GSE54236 cohort. **(G)** Kaplan–Meier overall survival analysis in GSE116174 cohort. **(H)** Kaplan–Meier overall survival analysis in GSE14520 cohort. **(I)** Heatmap and bar plot showing gene coefficients across different machine learning algorithms, illustrating the relative contribution of each gene to model performance.

Using multiple Machine Learning algorithms, we constructed prognostic models and comprehensively evaluated their performance based on the mean area under the (AUC) values for 1, 3, and 5-year survival (**Figure 2A**). Among these, the Elastin_net_0.2 model demonstrated the most robust predictive capacity, achieving consistently high mean AUC values across all three time points. Kaplan–Meier survival analyses across seven independent survival cohorts from TCGA, GSE54236, GSE116174, and GSE14520 (**Figures 2B–H**) consistently demonstrated that patients in the high-risk group exhibited significantly worse overall survival compared to those in the low-risk group. Furthermore, the heatmap of regression coefficients (**Figure 2I**) provided a clear visualization of the contribution of each input gene across different prognostic models, enabling an intuitive understanding of their relative importance in risk prediction.

### NOP56 overexpression predicts poor HCC outcomes

**Figure 3:**
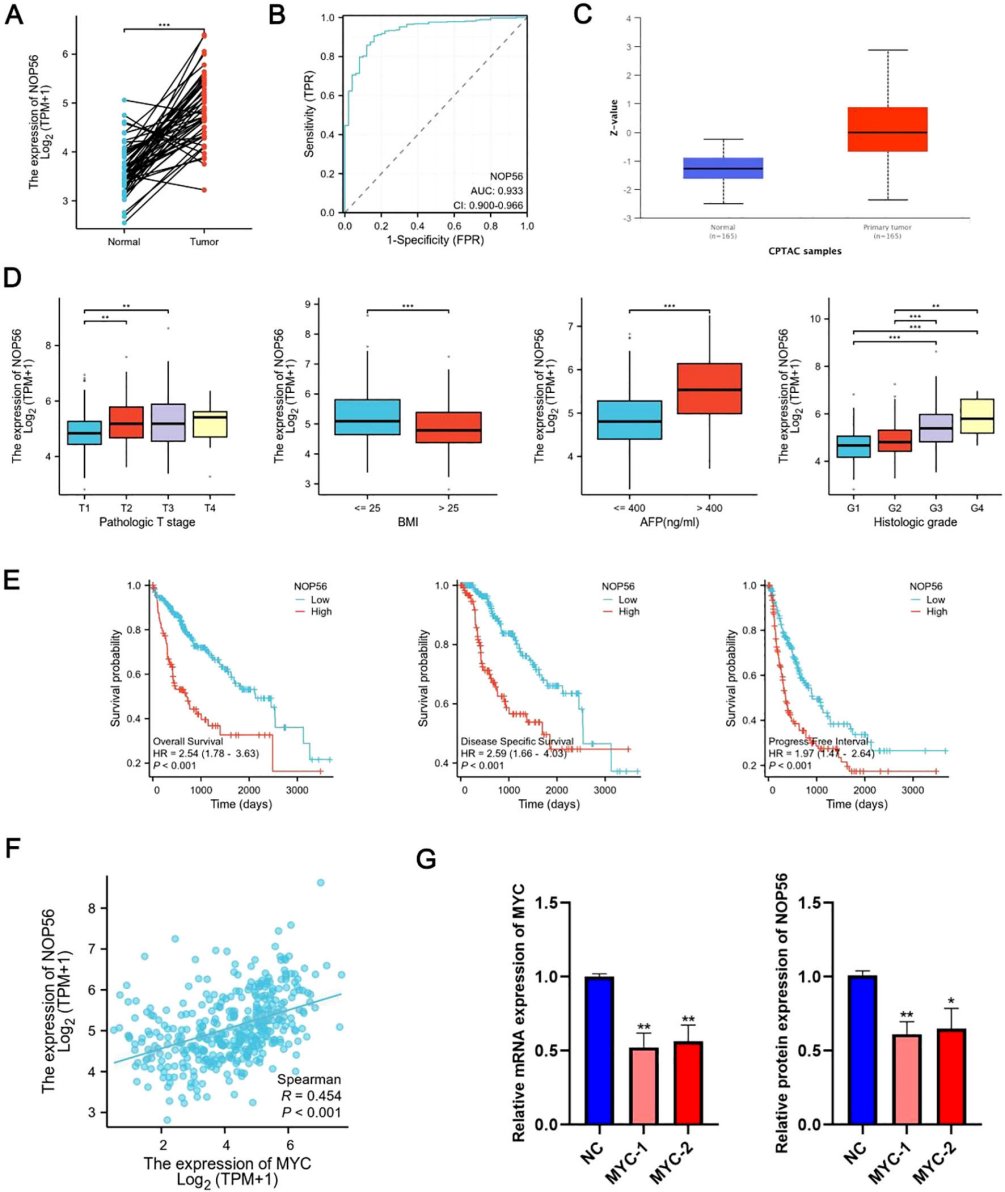
NOP56 is upregulated in HCC and correlates with tumor progression and poor prognosis. **(A)** Paired analysis of TCGA-HCC samples showing significantly higher NOP56 mRNA expression in tumors than in adjacent normal tissues. **(B)** ROC analysis demonstrates strong diagnostic performance of NOP56 in distinguishing HCC from normal liver. **(C)** CPTAC proteomics data confirming elevated NOP56 protein levels in HCC. **(D)** Association of NOP56 expressions with clinicopathological features, including T stage, BMI, AFP level, and histologic grade. **(E)** Kaplan–Meier curves indicating that high NOP56 expression is associated with worse OS, DSS, and PFI in the TCGA cohort. **(F)** Spearman analysis showing a significant positive correlation between MYC and NOP56 expression in TCGA-HCC samples. **(G)** Suppression of MYC using two independent MYC-targeting plasmids leads to reduced MYC and NOP56 mRNA levels, indicating MYC-dependent regulation of NOP56. Data were shown as mean ± SD. *: *p* < 0.05, **: *p* < 0.01, ***: *p* < 0.001.

Multiple studies have identified NOP56 as a potential prognostic marker in HCC (30–32). In this study, we first evaluated its clinical relevance. NOP56 expression was significantly higher in tumor tissues than in adjacent normal tissues in the TCGA cohort (**Figure 3A**). ROC curve analysis demonstrated a strong diagnostic value of NOP56 for distinguishing HCC from normal liver tissues (**Figure 3B**). Consistently, CPTAC proteomic data confirmed that NOP56 protein levels were markedly elevated in HCC samples (**Figure 3C**). Clinically, increased NOP56 expression was associated with more advanced pathological T stage, higher BMI, elevated AFP levels, and poorer histological differentiation (**Figure 3D**). Survival analyses further showed that patients with high NOP56 expression exhibited significantly shorter overall survival (OS), disease-specific survival (DSS), and progression-free interval (PFI) (**Figure 3E**), supporting its potential as an unfavorable prognostic biomarker. Given the oncogenic role of MYC, we next assessed its association with NOP56. TCGA analysis revealed a significant positive correlation between MYC and NOP56 expression (**Figure 3F**). Consistently, MYC knockdown using two independent plasmids reduced both MYC and NOP56 mRNA levels (**Figure 3G**), indicating that NOP56 upregulation may be at least partly driven by MYC signaling.

### Validation of NOP56 expression and nucleolar localization in HCC

**Figure 4:**
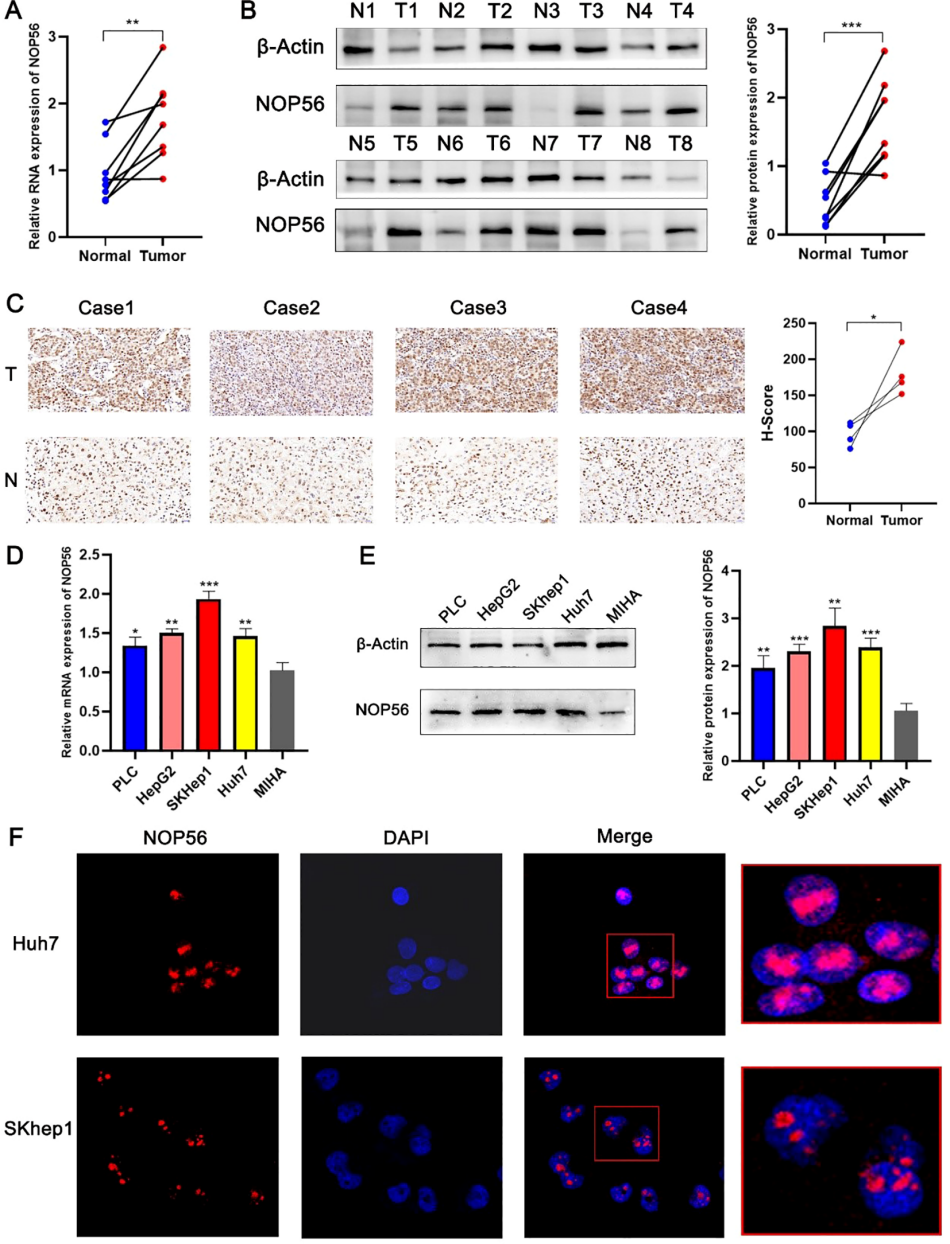
Validation of NOP56 expression and subcellular localization in HCC. **(A)** RT-PCR analysis of NOP56 mRNA levels in paired HCC and adjacent non-tumor tissues. **(B)** Western blot analysis of NOP56 protein expression in paired HCC and adjacent non-tumor tissues, with quantification on the right. **(C)** Representative immunohistochemical staining of NOP56 in HCC and adjacent non-tumor tissues, with H-score analysis. **(D)** NOP56 mRNA expression in liver cancer cell lines (PLC, HepG2, SKhep1, Huh7) and normal liver cell line (MIHA). **(E)** Western blot analysis of NOP56 protein expression in liver cancer cell lines and MIHA, with quantification on the right. **(F)** Immunofluorescence images showing predominant nucleolar localization of NOP56 in Huh7 and SKhep1 cells. Data were shown as mean ± SD. *: *p* < 0.05, **: *p* < 0.01, ***: *p* < 0.001.

Analysis of RNA expression profiles revealed significantly higher NOP56 transcript levels in tumor tissues (**Figure 4A**). Consistent with the RNA data, protein expression analysis demonstrated a marked upregulation of NOP56 in HCC tissues relative to controls (**Figure 4B**). Immunohistochemical staining further confirmed the elevated expression of NOP56 in tumor samples (**Figure 4C**). In liver cancer cell lines, NOP56 mRNA levels were significantly higher than in MIHA (**Figure 4D**), and similar trends were observed at the protein level (**Figure 4E**). Notably, across multiple platforms, including TCGA bulk RNA-seq, single-cell RNA-seq, and CPTAC proteomic datasets, NOP56 consistently exhibited tumor-specific overexpression, supporting the robustness of its dysregulated pattern in HCC. Immunofluorescence staining revealed a predominant nucleolar localization of NOP56 in HCC cells (**Figure 4F**).

### NOP56 knockdown inhibits HCC cell growth and metastasis *in vitro* and *in vivo*

**Figure 5:**
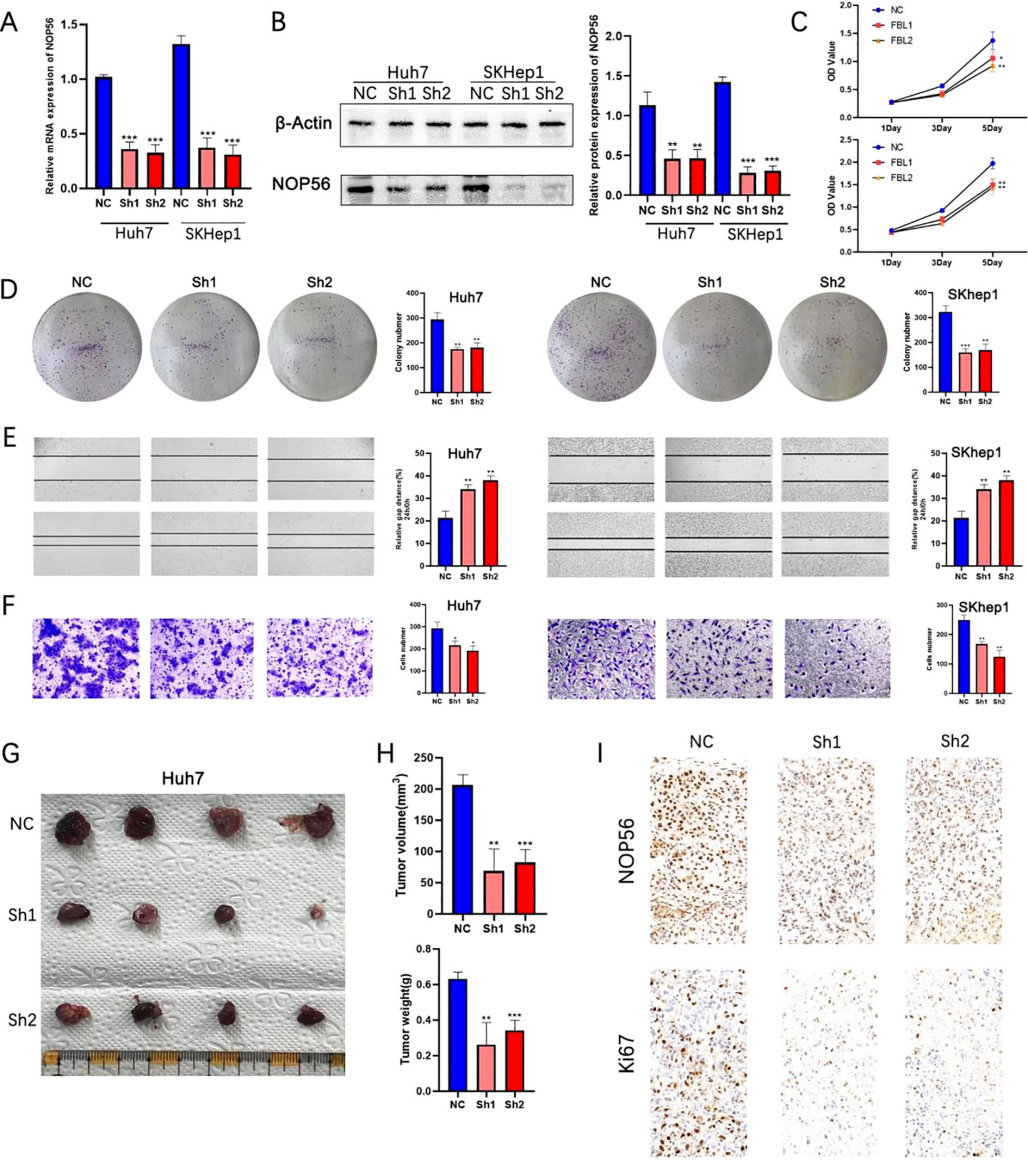
Silencing of NOP56 suppresses proliferation, migration, and tumorigenicity of HCC cells *in vitro* and *in vivo*. **(A)** qRT-PCR analysis of NOP56 mRNA levels in Huh7 and SKhep1 cells transduced with control (NC) or NOP56-targeting shRNAs (Sh1, Sh2). **(B)** Western blot analysis of NOP56 protein levels with β-actin as a loading control; quantification shown on the right. **(C)** Growth curves showing reduced proliferation in NOP56-knockdown cells compared with NC. **(D)** Representative images and quantification of colony formation assays. **(E)** Wound-healing assays showing impaired migration after NOP56 knockdown. **(F)** Transwell migration assays confirming reduced migratory capacity. **(G–H)** Representative xenograft tumors, tumor volume, and weight measurements from nude mice injected with Huh7 cells expressing NC or NOP56 shRNAs. **(I)** Immunohistochemical staining of NOP56 and Ki-67 in xenograft tumor tissues. Data were shown as mean ± SD. *: *p* < 0.05, **: *p* < 0.01, ***: *p* < 0.001.

To investigate the functional role of NOP56 in HCC, we established stable NOP56 knockdown HCC cell lines using lentivirus-mediated short hairpin RNAs (shRNAs). Given that Huh7 and Skhp1 exhibited the highest expression levels, we selected these two cell lines for subsequent functional experiments. Quantitative PCR confirmed that NOP56 mRNA expression was significantly reduced upon shRNA transduction (**Figure 5A**), and Western blot analysis further validated a consistent decrease at the protein level (**Figure 5B**). Cell growth assays revealed that knockdown of NOP56 led to a marked suppression in cell proliferation over time, as shown by reduced growth rates in both Huh7 and SKhep1 cells (**Figure 5C**). Consistently, colony formation assays demonstrated that the ability of cells to form colonies was significantly impaired following NOP56 silencing (**Figure 5D**), indicating decreased long-term proliferative capacity. In wound-healing assays, NOP56-depleted cells showed significantly reduced migration distance compared to controls, suggesting impaired migratory potential (**Figure 5E**). This was further confirmed by Transwell assays, in which the number of migrated cells was markedly reduced after NOP56 knockdown (**Figure 5F**). To assess the vivo relevance, subcutaneous xenografts were established in nude mice using Huh7 cells. NOP56 knockdown markedly reduced tumor volume and weight compared with controls (**Figures 5G,H**). Immunohistochemistry confirmed lower NOP56 expression and fewer Ki-67–positive cells in xenografts (**Figure 5I**). These findings indicate that NOP56 facilitates HCC proliferation, migration, and tumorigenesis, acting as a potential oncogenic effector downstream of C-myc.

### NOP56 regulates cell cycle progression and apoptosis in HCC cells

**Figure 6:**
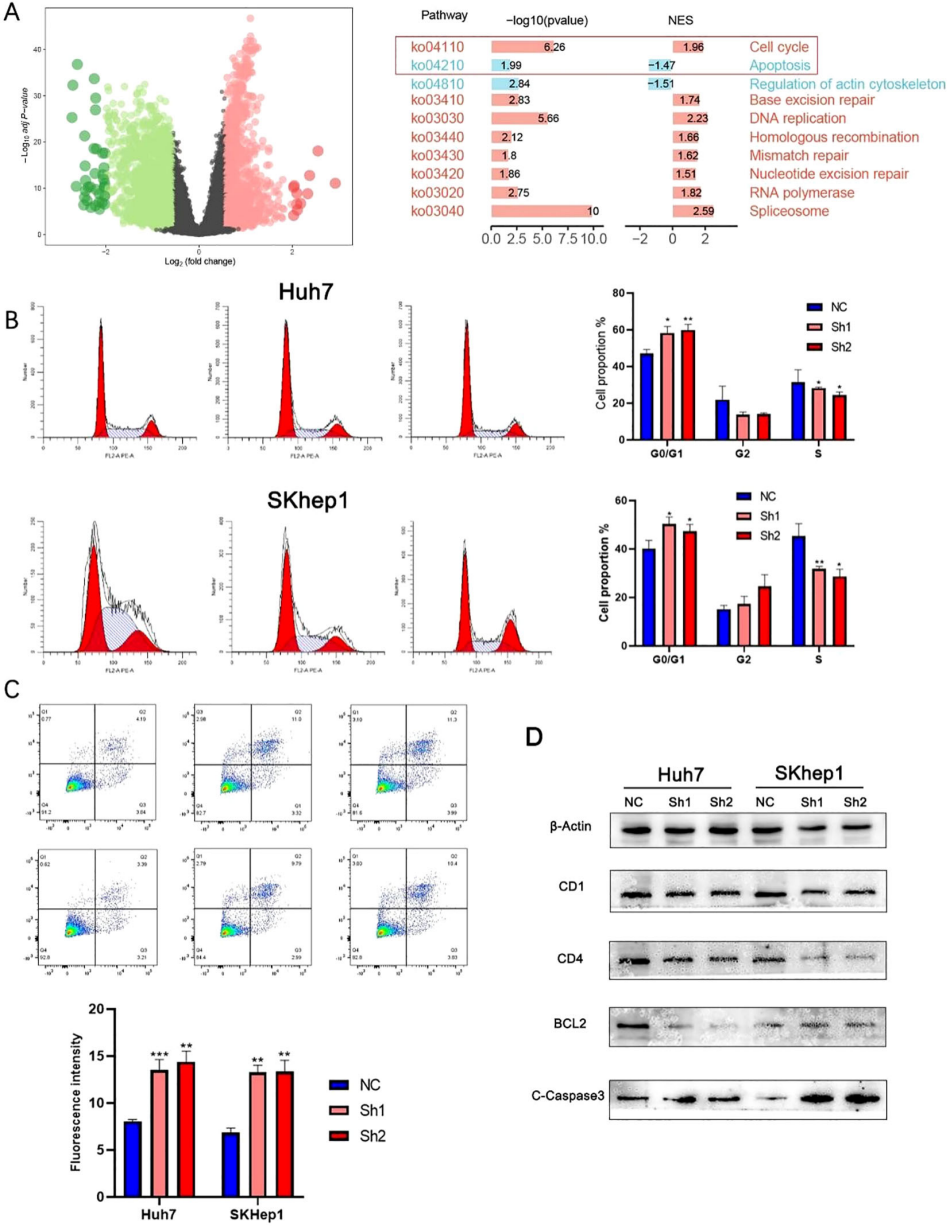
NOP56 regulates cell cycle progression and apoptosis in HCC cells. **(A)** Volcano plot of differentially expressed genes (DEGs) between high and low NOP56 expression groups in TCGA-LIHC; KEGG pathway enrichment analysis showing significant enrichment in cell cycle and apoptosis-related pathways. **(B)** Flow cytometry analysis of cell cycle distribution in Huh7 and SKhep1 cells with NOP56 knockdown, indicating G0/G1 arrest. **(C)** Annexin V/PI staining and quantification showing increased apoptosis after NOP56 silencing. **(D)** Western blot analysis of CDK1, CDK4, BCL2, and cleaved caspase-3 (C-Caspase3) expression in NOP56-depleted and control cells. Data were shown as mean ± SD. *: *p* < 0.05, **: *p* < 0.01, ***: *p* < 0.001.

Next, TCGA-LIHC tumors were stratified into high- and low-expression groups by median NOP56 level. Differential analysis with the “limma” package identified DEGs, which were significantly enriched in cell cycle and apoptosis-related pathways according to GO and KEGG analyses (**Figures 6A**). To confirm these results, cell cycle analysis after NOP56 knockdown in Huh7 and SKhep1 cells showed G0/G1 phase accumulation and reduced S-phase populations, indicating G1 arrest (**Figure 6B**). Annexin V/PI staining further demonstrated a significant increase in apoptosis in both cell lines (**Figure 6C**). Western blot analysis showed downregulation of the cell cycle regulators CDK1 and CD4, along with decreased expression of the anti-apoptotic protein BCL2 and increased cleaved caspase-3 (C-Caspase3), confirming the activation of apoptotic pathways following NOP56 suppression (**Figure 6D**). Collectively, these data suggest that NOP56 promotes tumor progression in HCC at least by facilitating cell cycle progression and suppressing apoptosis.

### NOP56 interacts with FBL and regulates the PI3K/AKT/CREB pathway to modulate apoptosis in HCC cells

**Figure 7:**
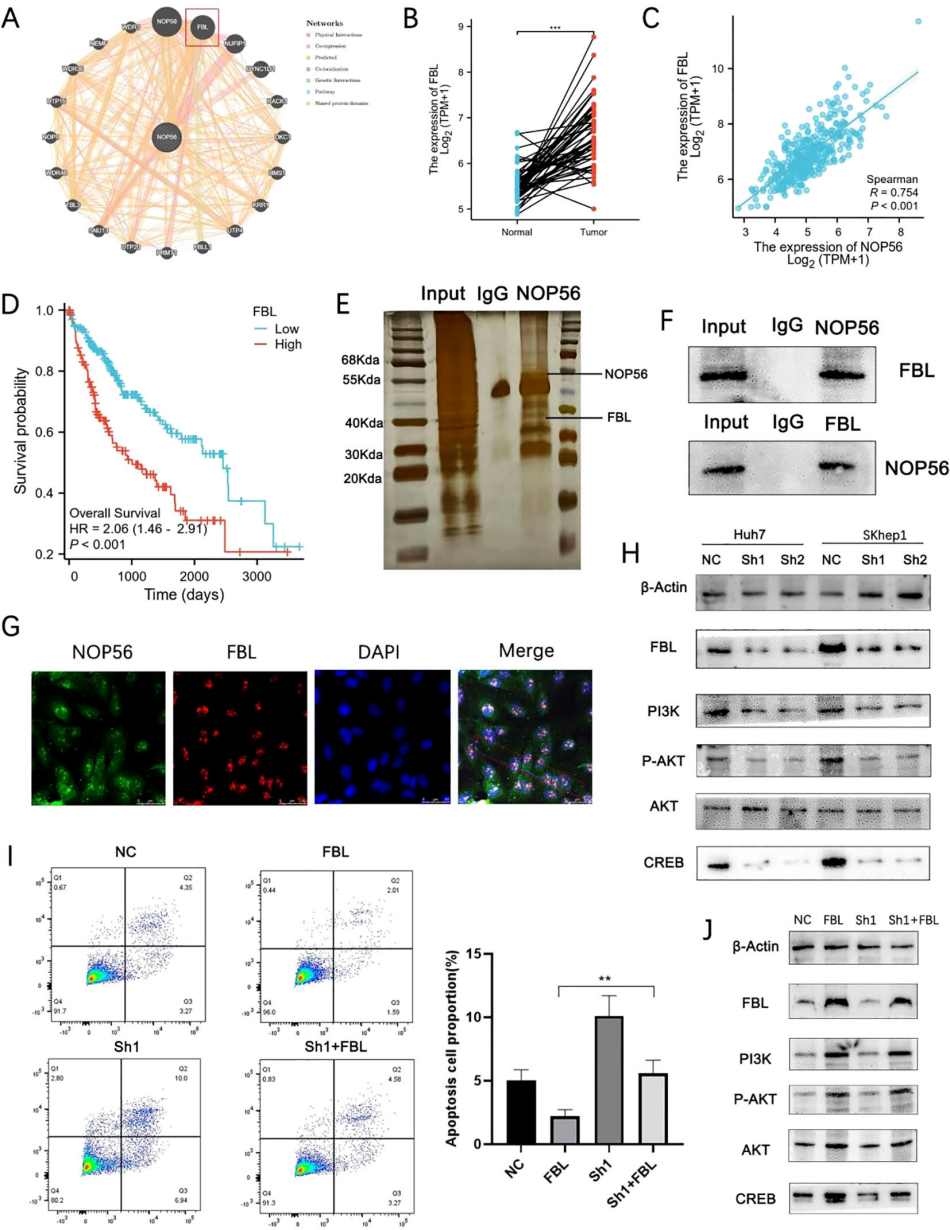
NOP56 interacts with FBL to promote HCC progression via the PI3K/AKT/CREB pathway. **(A)** Protein–protein interaction network predicted by GeneMANIA highlighting FBL as a strong interactor of NOP56. **(B)** FBL mRNA expression in TCGA-LIHC tumor versus normal tissues. **(C)** Spearman correlation between NOP56 and FBL expression. **(D)** Kaplan–Meier analysis showing poorer overall survival in patients with high FBL expression. **(E)** Silver staining of Co-IP products confirming FBL association with NOP56. **(F)** Reciprocal Co-IP followed by Western blot validating the interaction between NOP56 and FBL. **(G)** Immunofluorescence showing partial nucleolar co-localization of NOP56 and FBL. **(H)** Western blot showing decreased FBL and PI3K/AKT/CREB pathway components after NOP56 knockdown. **(I)** Annexin V/PI assay showing partial rescue of apoptosis in NOP56-silenced cells by FBL overexpression. **(J)** Western blot showing restoration of PI3K/AKT/CREB signaling after FBL overexpression in NOP56-knockdown cells. Data were shown as mean ± SD. *: *p* < 0.05, **: *p* < 0.01, ***: *p* < 0.001.

To explore how NOP56 promotes HCC progression, we analyzed its protein–protein interaction network via GeneMANIA. Fibrillarin (FBL), a key component of the nucleolar box C/D snoRNP complex, showed the strongest predicted association (**Figure 7A**). TCGA-LIHC data indicated that FBL was significantly upregulated in tumors (**Figure 7B**) and positively correlated with NOP56 expression (**Figure 6C**). Kaplan–Meier analysis linked high FBL expression to poor overall survival in HCC patients (**Figure 7D**), suggesting its oncogenic role. Co-immunoprecipitation and silver staining confirmed a physical interaction between NOP56 and FBL, further validated by reciprocal Western blotting (**Figures 7E, F**). Immunofluorescence revealed their partial co-localization in nucleoli (**Figure 7G**), indicating potential functional cooperation.

Given FBL’s role in ribosome biogenesis, oncogenic signaling, and 2′-O-methylation (Nm)–mediated regulation of the PI3K/AKT/mTOR pathway (33), we hypothesized that the NOP56–FBL axis may drive HCC progression via this signaling cascade. Western blot analysis showed that NOP56 knockdown reduced FBL levels and downregulated PI3K, phosphorylated AKT, and CREB in Huh7 and SKhep1 cells (**Figure 7H**). Rescue experiments demonstrated that ectopic FBL expression partially reversed NOP56 depletion–induced apoptosis (**Figure 7I**) and restored PI3K/AKT/CREB activation (**Figure 7J**) in SKHep1 cells. These findings indicate that NOP56 supports HCC cell survival partly through FBL-mediated activation of the PI3K/AKT/CREB pathway.

## Discussion

HCC is an aggressive cancer with limited therapies and poor prognosis, especially at intermediate or advanced stages (34). Despite recent advances in targeted therapy and immunotherapy, there remains an urgent need to uncover novel molecular mechanisms and therapeutic targets to improve patient outcomes (35, 36). In this study, we identified the nucleolar protein NOP56 as a novel oncogenic factor in HCC, promoting tumor growth by regulating proliferation, migration, cell cycle progression, and apoptosis. Using single-cell transcriptomics, bulk RNA-seq, and experimental validation, we confirmed its marked upregulation in HCC tissues and cell lines and its association with poor prognosis. Mechanistically, NOP56 interacts with the nucleolar protein FBL to activate the PI3K/AKT/CREB pathway, thereby suppressing apoptosis. These results reveal a previously unrecognized oncogenic role for nucleolar proteins in HCC and position the NOP56–FBL axis as a promising therapeutic target.

The nucleolus has increasingly been recognized as a regulatory hub for cancer progression, beyond its traditional role in ribosome biogenesis (37, 38). Dysregulation of nucleolar proteins such as NOP56 and FBL has been implicated in the development of various malignancies (25, 39). Our findings are consistent with previous reports that NOP56 is overexpressed in breast cancer (24) and Burkitt’s lymphoma (40), and extend its relevance to HCC. The strong association between NOP56 expression and aggressive clinical features, including advanced T stage, high AFP levels, and poor differentiation, suggests that NOP56 may complement existing biomarkers for early detection and prognostic stratification.

By performing differential gene expression and enrichment analysis, we found that NOP56 is closely associated with cell cycle progression and apoptosis-related pathways, which are known hallmarks of C-myc–driven tumorigenesis (41). Given that NOP56 is a direct transcriptional target of C-myc, as previously reported (40), our data support the hypothesis that aberrant activation of C-myc in HCC leads to upregulation of NOP56, thereby driving oncogenic signaling cascades. Functional assays confirmed that silencing NOP56 inhibits HCC cell proliferation and migration both *in vitro* assays and *in vivo* xenograft models, induces G0/G1 cell cycle arrest, and increases apoptosis, suggesting that NOP56 plays a critical role in maintaining malignant phenotypes.

A key mechanistic finding of our study is the identification of FBL as a functional partner of NOP56. As a core component of the box C/D snoRNP complex, FBL is indispensable for rRNA processing and 2′-O-methylation of target RNAs (26). FBL-mediated rRNA 2’-O-methylation promotes ribosome biogenesis and translational efficiency; dysregulation of this modification enhances oncogenic protein synthesis and has been increasingly recognized as a driver of cancer progression. Previous studies indicate that FBL can methylate oncogenic mRNAs such as PIK3CA, thereby enhancing their translation and stability (33). This provides a plausible mechanism through which the NOP56–FBL complex facilitates PI3K/AKT pathway activation. Consistently, we observed that NOP56 knockdown reduced FBL expression and suppressed PI3K/AKT/CREB signaling, while ectopic FBL expression partially reversed this effect. These results suggest that NOP56 stabilizes or cooperates with FBL to maintain PI3K/AKT activity and inhibit apoptosis in HCC. Notably, the NOP56–FBL interaction or their involvement in ribosome biogenesis-related oncogenic pathways has also been observed in other malignancies, such as colorectal cancer and breast cancer, suggesting that this regulatory axis may represent a more universal cancer-promoting mechanism rather than one restricted to HCC (42). Our data implies a regulatory dependency, whether the reduction of FBL upon NOP56 depletion is mediated through a direct molecular interaction or involves additional upstream regulatory factors remains to be fully elucidated and will be the subject of future mechanistic investigation.

The PI3K/AKT cascade is among the most activated oncogenic pathways in HCC, promoting survival, angiogenesis, and therapy resistance (43, 44). CREB, a downstream effector of AKT, drives anti-apoptotic genes such as BCL2 and survivin, further supporting tumor progression (45). Our findings indicate that NOP56 may facilitate HCC development by sustaining this pathway via FBL interaction. Targeting the NOP56–FBL axis to restore apoptosis could therefore provide a promising strategy to overcome drug resistance in HCC (46). At present, no small-molecule inhibitors specifically targeting NOP56 or FBL are available. However, nucleolar stress–inducing agents and RNA-modification inhibitors may potentially target this axis, providing a conceptual framework for therapeutic exploitation.

While our study provides compelling evidence for the oncogenic role of NOP56 and its interaction with FBL in HCC, several limitations should be acknowledged. First, although we validated the NOP56–FBL interaction using co-immunoprecipitation and NOP56 depletion significantly reduced FBL expression, it remains unclear whether this regulation is direct or mediated through downstream transcriptional or post-transcriptional factors. Further mechanistic studies are warranted. Second, the *in vivo* rescue experiments were limited to subcutaneous xenograft models; future studies employing orthotopic or PDX models would help confirm clinical relevance. Importantly, the broader transcriptomic effects of NOP56/FBL modulation remain to be characterized by RNA-seq or RIP-seq.

In conclusion, our study identifies NOP56 as an oncogenic nucleolar protein in HCC that drives tumor progression through interaction with FBL and activation of the PI3K/AKT/CREB pathway. This work enhances understanding of nucleolar protein networks in liver cancer and positions the NOP56–FBL axis as a potential therapeutic target.

## Data Availability

Gene transcriptome was obtained from The Cancer Genome Atlas (TCGA) (https://www.cancer.gov) for liver hepatocellular carcinoma (LIHC). Single-cell RNA sequencing (scRNA-seq) data were acquired from the Tumor Immune Single-cell Hub (TISCH) database (GSE166635). The original contributions presented in the study are included in the article/**Supplementary Material**, further inquiries can be directed to the corresponding author.

## References

[1] Vogel A Meyer T Sapisochin G Salem R Saborowski A . Hepatocellular carcinoma. Lancet (London England). (2022) 400:1345–62. doi: 10.1016/S0140-6736(22)01200-4, PMID: 36084663

[2] Siegel RL Kratzer TB Giaquinto AN Sung H Jemal A . Cancer statistics, 2025. CA: Cancer J Clin. (2025) 75:10–45. doi: 10.3322/caac.21871, PMID: 39817679 PMC11745215

[3] Ganesan P Kulik LM . Hepatocellular carcinoma: new developments. Clinics Liver Dis. (2023) 27:85–102. doi: 10.1016/j.cld.2022.08.004, PMID: 36400469

[4] Feng F Zhao Y . Hepatocellular carcinoma: prevention, diagnosis, and treatment. Med Principles Pract: Int J Kuwait Univ Health Sci Centre. (2024) 33:414–23. doi: 10.1159/000539349, PMID: 38772352 PMC11460940

[5] Hwang SY Danpanichkul P Agopian V Mehta N Parikh ND Abou-Alfa GK . Hepatocellular carcinoma: updates on epidemiology, surveillance, diagnosis and treatment. Clin Mol Hepatol. (2025) 31:S228–S54. doi: 10.3350/cmh.2024.0824, PMID: 39722614 PMC11925437

[6] Llovet JM Kelley RK Villanueva A Singal AG Pikarsky E Roayaie S . Hepatocellular carcinoma. Nat Rev Dis Primers. (2021) 7:6. doi: 10.1038/s41572-020-00240-3, PMID: 33479224 PMC13537433

[7] Garcia-Lezana T Lopez-Canovas JL Villanueva A . Signaling pathways in hepatocellular carcinoma. Adv Cancer Res. (2021) 149:63–101. doi: 10.1016/bs.acr.2020.10.002, PMID: 33579428

[8] Elhamamsy AR Metge BJ Alsheikh HA Shevde LA Samant RS . Ribosome biogenesis: A central player in cancer metastasis and therapeutic resistance. Cancer Res. (2022) 82:2344–53. doi: 10.1158/0008-5472.CAN-21-4087, PMID: 35303060 PMC9256764

[9] Zhou Y Gao X Yuan M Yang B He Q Cao J . Targeting myc interacting proteins as a winding path in cancer therapy. Front Pharmacol. (2021) 12:748852. doi: 10.3389/fphar.2021.748852, PMID: 34658888 PMC8511624

[10] Li J Zhu Y . Recent advances in liver cancer stem cells: non-coding RNAs, oncogenes and oncoproteins. Front Cell Dev Biol. (2020) 8:548335. doi: 10.3389/fcell.2020.548335, PMID: 33117795 PMC7575754

[11] Liu J Xu R Mai SJ Ma YS Zhang MY Cao PS . LncRNA CSMD1–1 promotes the progression of Hepatocellular Carcinoma by activating MYC signaling. Theranostics. (2020) 10:7527–44. doi: 10.7150/thno.45989, PMID: 32685003 PMC7359090

[12] Xia P Zhang H Xu K Jiang X Gao M Wang G . MYC-targeted WDR4 promotes proliferation, metastasis, and sorafenib resistance by inducing CCNB1 translation in hepatocellular carcinoma. Cell Death Dis. (2021) 12:691. doi: 10.1038/s41419-021-03973-5, PMID: 34244479 PMC8270967

[13] Wang H Zhang S Zhang Y Jia J Wang J Liu X . TAZ is indispensable for c-MYC-induced hepatocarcinogenesis. J Hepatol. (2022) 76:123–34. doi: 10.1016/j.jhep.2021.08.021, PMID: 34464659 PMC9569156

[14] Dhanasekaran R Deutzmann A Mahauad-Fernandez WD Hansen AS Gouw AM Felsher DW . The MYC oncogene - the grand orchestrator of cancer growth and immune evasion. Nat Rev Clin Oncol. (2022) 19:23–36. doi: 10.1038/s41571-021-00549-2, PMID: 34508258 PMC9083341

[15] Lourenco C Resetca D Redel C Lin P Macdonald AS Ciaccio R . MYC protein interactors in gene transcription and cancer. Nat Rev Cancer. (2021) 21:579–91. doi: 10.1038/s41568-021-00367-9, PMID: 34188192

[16] Zeng Y Jiang H Zhang X Xu J Wu X Xu Q . Canagliflozin reduces chemoresistance in hepatocellular carcinoma through PKM2-c-Myc complex-mediated glutamine starvation. Free Radical Biol Med. (2023) 208:571–86. doi: 10.1016/j.freeradbiomed.2023.09.006, PMID: 37696420

[17] Her J Zheng H Bunting SF . RNF4 sustains Myc-driven tumorigenesis by facilitating DNA replication. J Clin Invest. (2024) 134. doi: 10.1172/JCI167419, PMID: 38530355 PMC11093604

[18] Moon H Park H Ro SW . c-myc-driven hepatocarcinogenesis. Anticancer Res. (2021) 41:4937–46. doi: 10.21873/anticanres.15307, PMID: 34593441

[19] Lacroix E Momchilova EA Chandhok S Padavu M Zapf R Audas TE . PI3K/AKT signaling mediates stress-inducible amyloid formation through c-Myc. Cell Rep. (2025) 44:115617. doi: 10.1016/j.celrep.2025.115617, PMID: 40272983

[20] Gautier T Bergès T Tollervey D Hurt E . Nucleolar KKE/D repeat proteins Nop56p and Nop58p interact with Nop1p and are required for ribosome biogenesis. Mol Cell Biol. (1997) 17:7088–98. doi: 10.1128/MCB.17.12.7088, PMID: 9372940 PMC232565

[21] Zhao S Zhang D Liu S Huang J . The roles of NOP56 in cancer and SCA36. Pathol Oncol Research: POR. (2023) 29:1610884. doi: 10.3389/pore.2023.1610884, PMID: 36741964 PMC9892063

[22] Ciribilli Y Singh P Inga A Borlak J . c-Myc targeted regulators of cell metabolism in a transgenic mouse model of papillary lung adenocarcinoma. Oncotarget. (2016) 7:65514–39. doi: 10.18632/oncotarget.11804, PMID: 27602772 PMC5323172

[23] Marcel V Catez F Berger CM Perrial E Plesa A Thomas X . Expression profiling of ribosome biogenesis factors reveals nucleolin as a novel potential marker to predict outcome in AML patients. PloS One. (2017) 12:e0170160. doi: 10.1371/journal.pone.0170160, PMID: 28103300 PMC5245884

[24] Su H Xu T Ganapathy S Shadfan M Long M Huang TH . Elevated snoRNA biogenesis is essential in breast cancer. Oncogene. (2014) 33:1348–58. doi: 10.1038/onc.2013.89, PMID: 23542174

[25] Yang Z Liang SQ Zhao L Yang H Marti TM Hegedüs B . Metabolic synthetic lethality by targeting NOP56 and mTOR in KRAS-mutant lung cancer. J Exp Clin Cancer Research: CR. (2022) 41:25. doi: 10.1186/s13046-022-02240-5, PMID: 35039048 PMC8762933

[26] Shubina MY Musinova YR Sheval EV . Nucleolar methyltransferase fibrillarin: evolution of structure and functions. Biochem Biokhimiia. (2016) 81:941–50. doi: 10.1134/S0006297916090030, PMID: 27682166

[27] Shubina MY Musinova YR Sheval EV . Proliferation, cancer, and aging-novel functions of the nucleolar methyltransferase fibrillarin? Cell Biol Int. (2018) 42:1463–6. doi: 10.1002/cbin.11044, PMID: 30080298

[28] Tao S Cui D Cheng H Liu X Jiang Z Chen H . High expression of TBRG4 in relation to unfavorable outcome and cell ferroptosis in hepatocellular carcinoma. BMC Cancer. (2024) 24:194. doi: 10.1186/s12885-024-11943-1, PMID: 38347489 PMC10860303

[29] Meng Y Zhao Q An L Jiao S Li R Sang Y . A TNFR2-hnRNPK axis promotes primary liver cancer development via activation of YAP signaling in hepatic progenitor cells. Cancer Res. (2021) 81:3036–50. doi: 10.1158/0008-5472.CAN-20-3175, PMID: 33619115

[30] Zhang Y Lin Z Lin X Zhang X Zhao Q Sun Y . A gene module identification algorithm and its applications to identify gene modules and key genes of hepatocellular carcinoma. Sci Rep. (2021) 11:5517. doi: 10.1038/s41598-021-84837-y, PMID: 33750838 PMC7943822

[31] Karaosmanoğlu O . Recurrent hepatocellular carcinoma is associated with the enrichment of MYC targets gene sets, elevated high confidence deleterious mutations and alternative splicing of DDB2 and BRCA1 transcripts. Adv Med Sci. (2025) 70:17–26. doi: 10.1016/j.advms.2024.10.004, PMID: 39486583

[32] Zhou W Chen Y Luo R Li Z Jiang G Ou X . Identification of biomarkers related to immune cell infiltration in hepatocellular carcinoma using gene co-expression network. Pathol Oncol Research: POR. (2021) 27:601693. doi: 10.3389/pore.2021.601693, PMID: 34257558 PMC8262220

[33] Wu W Chen X Liu X Bao HJ Li QH Xian JY . SNORD60 promotes the tumorigenesis and progression of endometrial cancer through binding PIK3CA and regulating PI3K/AKT/mTOR signaling pathway. Mol Carcinogenesis. (2023) 62:413–26. doi: 10.1002/mc.23495, PMID: 36562475

[34] Abdelhamed W El-Kassas M . Hepatocellular carcinoma recurrence: Predictors and management. Liver Res (Beijing China). (2023) 7:321–32. doi: 10.1016/j.livres.2023.11.004, PMID: 39958776 PMC11791921

[35] Bicer F Kure C Ozluk AA El-Rayes BF Akce M . Advances in immunotherapy for hepatocellular carcinoma (HCC). Curr Oncol (Toronto Ont). (2023) 30:9789–812. doi: 10.3390/curroncol30110711, PMID: 37999131 PMC10670350

[36] Sangro B Sarobe P Hervás-Stubbs S Melero I . Advances in immunotherapy for hepatocellular carcinoma. Nat Rev Gastroenterol Hepatol. (2021) 18:525–43. doi: 10.1038/s41575-021-00438-0, PMID: 33850328 PMC8042636

[37] Stępiński D . The nucleolus, an ally, and an enemy of cancer cells. Histochem Cell Biol. (2018) 150:607–29. doi: 10.1007/s00418-018-1706-5, PMID: 30105457 PMC6267422

[38] Montanaro L Treré D Derenzini M . Nucleolus, ribosomes, and cancer. Am J Pathol. (2008) 173:301–10. doi: 10.2353/ajpath.2008.070752, PMID: 18583314 PMC2475768

[39] Yang L Zhang Z Jiang P Kong D Yu Z Shi D . Phase separation-competent FBL promotes early pre-rRNA processing and translation in acute myeloid leukaemia. Nat Cell Biol. (2024) 26:946–61. doi: 10.1038/s41556-024-01420-z, PMID: 38745030

[40] Cowling VH Turner SA Cole MD . Burkitt’s lymphoma-associated c-Myc mutations converge on a dramatically altered target gene response and implicate Nol5a/Nop56 in oncogenesis. Oncogene. (2014) 33:3519–27. doi: 10.1038/onc.2013.338, PMID: 24013231 PMC5003617

[41] Llombart V Mansour MR . Therapeutic targeting of “undruggable” MYC. EBioMedicine. (2022) 75:103756. doi: 10.1016/j.ebiom.2021.103756, PMID: 34942444 PMC8713111

[42] Yi Y Li Y Meng Q Li Q Li F Lu B . A PRC2-independent function for EZH2 in regulating rRNA 2’-O methylation and IRES-dependent translation. Nat Cell Biol. (2021) 23:341–54. doi: 10.1038/s41556-021-00653-6, PMID: 33795875 PMC8162121

[43] Tian LY Smit DJ Jücker M . The role of PI3K/AKT/mTOR signaling in hepatocellular carcinoma metabolism. Int J Mol Sci. (2023) 24. doi: 10.3390/ijms24032652, PMID: 36768977 PMC9916527

[44] Bang J Jun M Lee S Moon H Ro SW . Targeting EGFR/PI3K/AKT/mTOR signaling in hepatocellular carcinoma. Pharmaceutics. (2023) 15. doi: 10.3390/pharmaceutics15082130, PMID: 37631344 PMC10458925

[45] Liang H Tang LY Ge HY Chen MM Lu SY Zhang HX . Neuronal survival factor TAFA2 suppresses apoptosis through binding to ADGRL1 and activating cAMP/PKA/CREB/BCL2 signaling pathway. Life Sci. (2023) 334:122241. doi: 10.1016/j.lfs.2023.122241, PMID: 37944639

[46] Ladd AD Duarte S Sahin I Zarrinpar A . Mechanisms of drug resistance in HCC. Hepatol (Baltimore Md). (2024) 79:926–40. doi: 10.1097/HEP.0000000000000237, PMID: 36680397

