# Supplementary material for: NOP56 interacts with Fibrarin to regulate the PI3K/AKT signaling pathway and inhibit apoptosis of hepatocellular carcinoma
Source: Front Oncol. 2026 Jan 6;15:1728226. doi: 10.3389/fonc.2025.1728226 (PMC12815711; doi:10.3389/fonc.2025.1728226)
Supplement: Supplementary file 1 [file DataSheet1.pdf]

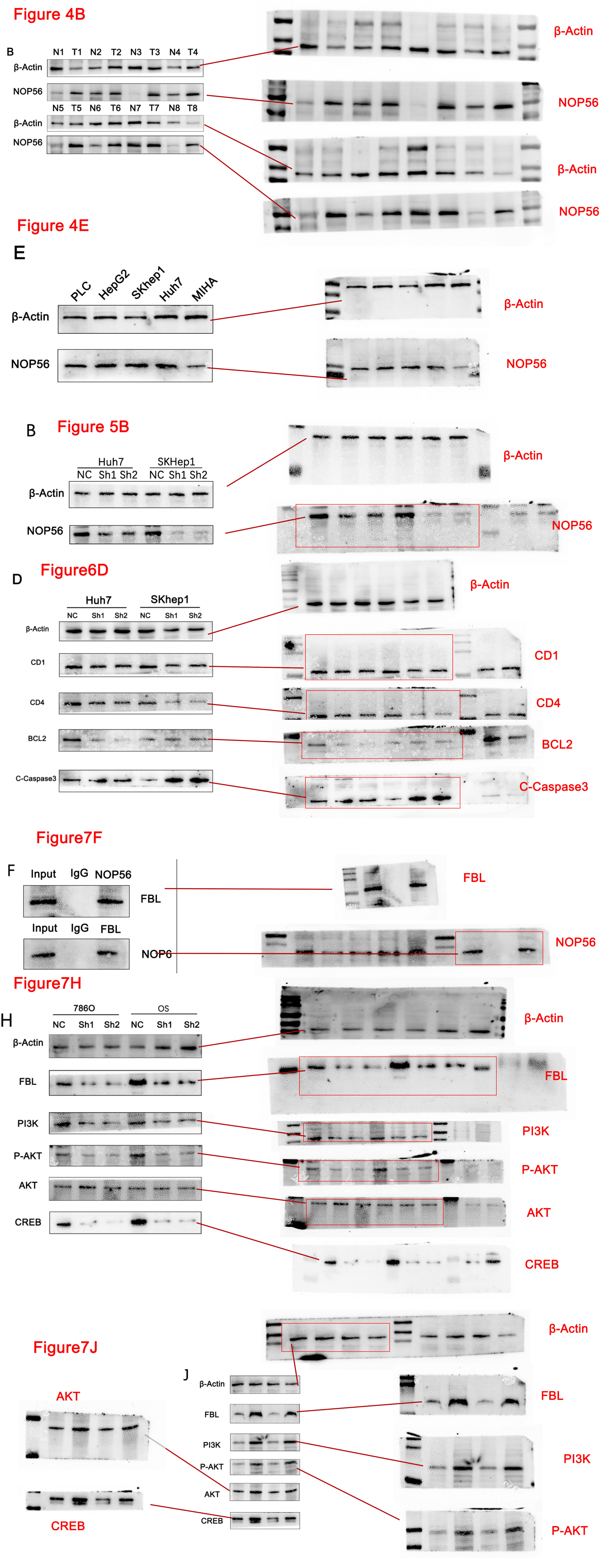

To reduce antibody usage, PVDF membranes were cut horizontally according to the molecular weight of the target proteins prior to antibody incubation. Each membrane strip was incubatedwith the appropriate primary antibody specific to its target protein.
